# General practice in the German Democratic Republic (1949–1990): A discipline between threat and professionalization

**DOI:** 10.1007/s00508-022-02093-0

**Published:** 2022-10-26

**Authors:** Florian Bruns, Christian König, Thomas Frese, Jan Schildmann

**Affiliations:** 1 Institut für Geschichte der Medizin, Medizinische Fakultät Carl Gustav Carus, Technische Universität Dresden, Fetscherstr. 74, 01307 Dresden, Germany; 2 Institut für Geschichte und Ethik der Medizin, Profilzentrum Gesundheitswissenschaften, Medizinische Fakultät, Martin-Luther-Universität Halle-Wittenberg, Halle (Saale), Germany; 3 Institut für Allgemeinmedizin, Profilzentrum Gesundheitswissenschaften, Medizinische Fakultät, Martin-Luther-Universität Halle-Wittenberg, Halle (Saale), Germany

**Keywords:** General practice, East Germany, History of medicine, Medical societies, Policlinics

## Abstract

**Background:**

In the 1950s the socialist health policy in East Germany did not follow a clear-cut course with regard to outpatient medical care. Whilst state-run policlinics gradually took the place of doctors in private practice, the required qualifications of physicians working in outpatient care remained unclear. After preparatory lobbying by committed physicians from the outpatient sector, the 1960 Weimar Health Conference finally paved the way for the preservation and professionalization of general practice in East Germany.

**Aim:**

The article analyzes the formation of general practice as a specialty in East Germany between 1945 and 1990. We scrutinize the status of general practitioners and their field in the socialist health system as well as the foundation of their medical society. Our paper aims to contribute to a broader history of general practice in Germany.

**Methods:**

We draw on literature from that time, unpublished archival material, and interviews with contemporary witnesses.

**Results:**

After the establishment of standards for specialist training in the early 1960s, general practice was introduced as a field of specialty in 1967. By this, East Germany had a compulsory specialist training in general practice much earlier than West Germany. In 1971, a specialist society for general practice was founded in East Germany. However, institutionalization at the medical faculties was still lacking. Meanwhile, the nationalization of outpatient care continued. In the years that followed, primary medical care was increasingly provided in policlinics. In 1989, of 40,000 physicians in the GDR, only about 340 were still practicing in their own offices.

**Conclusion:**

Within the nationalized GDR health system a committed group of physicians, under difficult political circumstances, pushed for professionalization of general practice and its recognition as a field of specialty. When general medicine was recognized as a specialty in 1967, this happened earlier than in other countries and constituted an important milestone.

## Introduction

Today, general practitioners (GPs) play a fundamental role in the care of the German population. Germans are frequent attenders of primary care services and the 44,000 GPs that are licensed in the country handle the majority of these patient contacts. GPs are “the first port of call” for “patients with all kinds of health problems”, as the German College of General Practitioners and Family Physicians puts it in a basic resolution [1]. As a cornerstone of the health system general practice is receiving a great deal of attention in German politics, the media and society. There is a chair for general practice at almost every medical faculty at German universities and the subject is increasingly represented in the medical school curriculum. However, a look into the past shows that general practice in Germany had to fight hard for its recognition as a specialty. In Germany, as in other countries, the professionalization of general practice took place in the decades after 1945, which was exactly the time when the country and its health system were divided in two antagonist parts (1949–1990). Thus, the development of general practice proceeded differently in East and West Germany. Interesting enough, in East Germany, i.e. the German Democratic Republic (GDR), the formation of general practice as a specialty took place earlier than in West Germany. In the GDR the general practitioner (*Facharzt für Allgemeinmedizin*) was introduced as a specialist designation in 1967. General practice, for the first time in Germany, became a medical specialty, requiring 5 years of postgraduate training and a board certification. It was not until 25 years later that the Federal Republic of Germany, then already a reunified country, reached this standard.^1^ Until now little is known about the emergence of general practice as a medical and academic discipline in divided Germany. In this article we focus on the formation of general practice as a specialty in East Germany. We scrutinize the status of general practitioners and their field in the GDR health system as well as the foundation of their medical society in 1971. There is sparse research literature on these topics [2–6]. We draw primarily on literature from the GDR era, unpublished archival material from the National Archives of Germany (*Bundesarchiv*) in Berlin, and interviews with contemporary witnesses. Our paper aims to contribute to a broader history of general practice in Germany, a story that has yet to be written.

## The transformation of outpatient care in the Soviet Occupation Zone and GDR

The socialist transformation of the East German healthcare system had begun as early as 1945. It was initiated by socialist health politicians who had been active in the Weimar Republic between 1919 and 1933. Soviet occupation forces and the ruling Socialist Unity Party (SED) supported and secured the restructuring. In order to prevent greater resistance from the medical profession the SED did not allow any medical lobby group to emerge. Regional medical associations, which had been banned by the Soviets in 1945 because of their Nazi affinity, were never permitted again in the GDR. The socialist healthcare concept was based on ideas developed in pre-fascist Germany on the one hand and principles of the Soviet health system on the other [7]. Health protection was defined as a public obligation, organized first and foremost by the government and detached from any commercial considerations. In order to strengthen non-profit healthcare the SED regime began nationalizing all major healthcare facilities, with the exception of denominational hospitals. Outpatient care was also affected by the socialist realignment. Since office-based physicians in particular were regarded as exponents of commercial thinking in medicine (specialists as well as nonspecialists), the government made efforts to reduce their number. From the late 1940s on, the establishment of physicians in private practice was strongly restricted. According to the Soviet model, state-run policlinics and ambulatory clinics (i.e. smaller versions of policlinics with only one or two doctors and often a dentist) should take the place of office-based physicians. In the GDR, as in the Soviet Union and Eastern Europe, a policlinic (*Poliklinik*) was an outpatient health care center funded by the government that provided both medical treatment and preventive healthcare. The prefix “poli-” derived from the Greek word *polis* (city) but also contained the meaning of *poly* (many), since by definition, policlinics were staffed with physicians from several specialties. In the GDR, minimum staffing consisted of an internist, a surgeon, a gynecologist, and a pediatrician.

In rural areas policlinics as well as ambulatory clinics were often set up in expropriated castles or manor houses, symbolizing the end of all feudalism under the new socialist order. In these newly formed healthcare centers physicians worked as modestly paid employees, a fact that the SED described as “democratization” of the health system [8]. The few doctors who were still allowed to set up their own practice bought this with disadvantages, for example a lower old-age pension. Already existing doctor’s offices were tolerated until the owner retired. Others were directly transformed into state-run institutions with the former owner as employee. In some cases, however, the authorities accepted succession arrangements within the family, i.e. physicians were allowed to hand over their practice to their children [3]. Without these concessions healthcare could not have been maintained, since in the 1950s office-based doctors were still a pillar of medical care in large parts of the GDR. In 1955, a little more than 5000 physicians were still working in their own practices, thus a good third of the 13,750 physicians registered in East Germany. Many of them rejected the idea of working in a policlinic since they failed to see any financial incentive. However, 5 years later the number of office-based doctors had fallen to 3250, while the total number of physicians had increased slightly to 14,550. Meanwhile, the number of policlinics had increased from 369 in 1955 to 403 in 1960 [9]. A year later, the SED regime built the Berlin Wall, thereby cementing the ideological and physical separation of the two German states. The Wall closed the last gap in the iron curtain and ended a mass exodus from the GDR that had become a threat to its existence. Between 1949 and 1961 almost 3 million people had left East Germany to live in the West, including several thousand physicians and dentists [10]. The resulting shortage of doctors remained noticeable in the GDR until 1990.

## Outpatient doctors under pressure

**Fig. 1:**
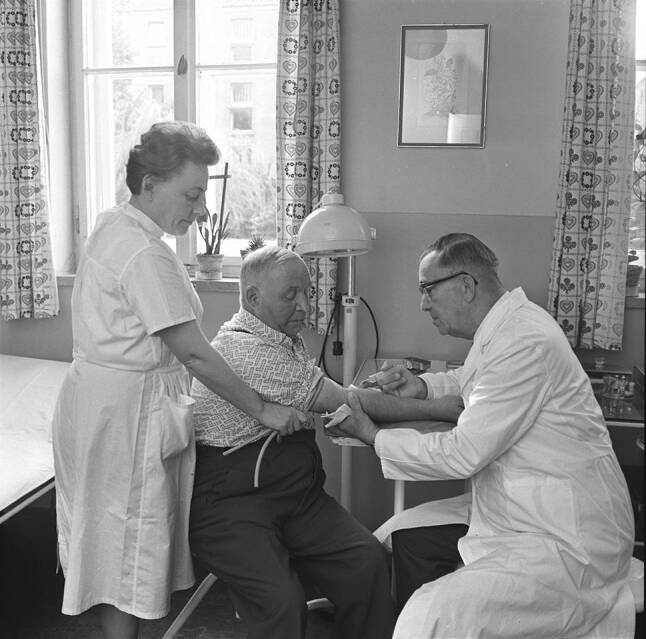
A rural outpatient clinic (*Landambulatorium*) in Berlstedt, a village in Thuringia. Dr. Köhler, head of the facility, is taking a patient’s blood sample (October 1967) (Bundesarchiv, Bild 183-F1010-0014-001/Fotograf: Demme, Dieter)

For decades, so-called practical doctors (*praktische Ärzte*) had borne the main burden of outpatient care in Germany. Practical doctors used to work in their own offices without specialization or board certification, since general practice had never been a medical specialty until the postwar era. Accordingly, the today common term general practitioner (*Facharzt für Allgemeinmedizin*) in the sense of a specialist was not in use; however, practical doctors had a wealth of experience. In rural areas, for example, they also often acted as obstetricians or pediatricians. In the midst of the socialist transformation of the East German health system these practical doctors in particular saw their status called into question. The SED’s aversion to self-employed persons and the growing number of state-run policlinics began to threaten their existence. In fact, the socialist propaganda proclaimed a new type of physician who would be part of a socialist collective in a policlinic and would no longer work on his own account. Some socialist healthcare theorists, like Karlheinz Renker (1921–1982), a social hygienist at Halle University, and Kurt Winter (1910–1987), likewise a social hygienist at Berlin University, actually sought to abolish practical doctors altogether. In a 1958 article in the main medical journal of the GDR Renker and Winter considered them superfluous if not harmful for patients [11]. The ostensible argument they put forward was the lack of qualifications among practical doctors who, like in many other countries at that time, practiced in a field without postgraduate specialization. In fact, this was an attempt to implement Soviet health policy in East Germany. In line with the Soviet model, Renker and Winter called for healthcare led by hospitals and a primary care concept that consisted of a minor qualified internist, the “therapewt” [3]. However, replacing an allegedly underqualified doctor with a barely better qualified internist appeared contradictory. Thus, in the ensuing debate a group of committed country doctors managed to fend off the implementation of what they called “barefoot doctors, Soviet style”.^2^ Heinz Brandt, an eloquent practical doctor from rural Saxony, was the first to publicly object against Renker’s and Winter’s claims. According to Brandt, patients in rural areas would rarely call a doctor because of a special disease but rather because of unspecific symptoms. Brandt stated that a well-trained practical doctor would be best suited for such cases (Fig. 1). He also expressed irritation that the contribution of practical doctors to overcome the postwar health crisis was so little appreciated by Renker and Winter [12]. Years later Brandt recalled his trepidation when submitting his critical response to the journal, all the more as he was not a member of the ruling SED [2]. Publicly disagreeing with two influential protagonists of the healthcare system (the fierce communist Winter in particular was not only engaged in academics but also in health policy) was indeed quite courageous in a political system that eagerly suppressed and discriminated against dissenters.

It quickly turned out that Brandt was not alone in his criticism. His article was followed by others who called for a better and institutionalized training for the group of practical doctors out in the country instead of abolishing them [13, 14]. Other authors criticized the concentration of health services at policlinics in general. This would lead to long access routes for patients and overcrowded waiting rooms. Treatment by changing physicians in the policlinic would also disrupt the doctor-patient relationship, as patients would miss their personal physician [15]. Shortly after Renker’s and Winter’s article appeared, all 15 regional district physicians (*Bezirksärzte*) of the GDR had already spoken out against the abolition of the practical doctors at a meeting in late 1958 [16]. Brandt and his colleagues also benefited from the fact that after the hype about Pawlow enthusiasm for the Soviet model generally waned in the GDR in the late 1950s. Concerns about losing even more doctors to West Germany also prevented the SED from further confronting the medical profession.

In 1960, a national health conference was convened in Weimar to stabilize and advance the East German health system and to counteract the ongoing flight of physicians. An important aim of the health conference was to send a signal to office-based physicians out in the country that their work was noticed and recognized by the SED. Already in his opening speech, Kurt Hager (1912–1998), a high-ranking functionary of the SED and the most politically powerful player at the conference, emphasized the important role of the practical doctors in the GDR healthcare system. Thus, it was already clear at the beginning of the conference that any plans to abolish practical doctors had been buried [17]. On the contrary, health officials put practical doctors on an equal footing with specialists and thus agreed to their central demand. In 1961, the Ministry of Health presented the first educational standard for specialist training to become a practical doctor and 3 years of postgraduate training were required to obtain the certificate. At regional district level, experienced practical doctors together with colleagues from other specialties formed commissions to set the learning objectives and conduct the examinations [18]. Practical doctors could finally appear more self-confident and feel accepted by the state. However, it was also clear that further progress could not be achieved against the socialist health policy or against the establishment of further policlinics. Professional independence was also limited, as all doctors were subject to the disciplinary authority of the municipal district physician (*Kreisarzt*). This subordination ensured the political and governmental influence of both the SED and the Ministry of Health. Their control could reach down to virtually every doctor’s office.

## Consolidation of general practice as a medical specialty

During the 1960s, general practice in East Germany was on its way to further professionalization. Strongly committed doctors, often from small towns or villages, who had come together in the regional examination commissions formed the spearhead of this movement. They received support from Herbert Knabe (1918–2009) who held the newly established chair for “Hygiene in the countryside” (*Hygiene auf dem Lande*) at Greifswald University since 1961. Oriented to the broader concept of hygiene that was typical for a socialist health system, Knabe and his team also researched on rural health care and country medicine. He thereby created one of the first and rare academic footholds of general practice in the GDR. Knabe and his team tirelessly published scientific articles, held symposia, and organized numerous courses in further training. These one‑week courses to upskill country doctors had been held annually since 1964 with well over 100 participants each year [19]. The Academy of Postgraduate Medical Education (*Akademie für Ärztliche Fortbildung*), founded in 1961 in East Berlin, represented another institutional anchor for general practice in the GDR. In 1967, the academy established two chairs of general practice. Since the academy was responsible for the continuing education and training of physicians, setting up these chairs was a prerequisite for the establishment of an official specialist training program in general practice [6]. In the same year, this specialist training program came into being: In the course of rewriting the educational and auditing standards for medical specializations the designation “practical doctor” was replaced by “general practitioner” (*Facharzt für Allgemeinmedizin*). This represented an important milestone. General practice, for the first time in Germany, became an official specialty, now requiring 5 years of postgraduate training and a board certification. The residency was restructured and systematized. The centralized structure of the GDR healthcare system facilitated the rapid nationwide implementation of the new training concept. From now on, further training ran under the aegis of the Academy of Postgraduate Medical Education, just as any postdoctoral qualification (“Promotion B” in GDR terminology) in general practice. The specialist training of GPs also included ideological and political teaching content, since the knowledge about the Marxist-Leninist philosophy and the socialist society was deemed necessary for doctors who were in such close contact with working-class people as GPs often are.

In the mid-1980s, signs of a crisis in primary care became increasingly apparent. Although expenditures increased, quality of care and patient satisfaction declined. More and more districts reported a shortage of GPs and difficulties in filling vacancies in policlinics and ambulatory clinics. To improve the situation, the SED leadership issued a resolution in November 1987 to strengthen the field of general practice (*Hausarztbeschluss*). A first starting point was to steer more graduates into general practice. By 1989, one third of the medical graduates of a year were directed into general practice residency programs.^3^ Apart from increasing the number of GPs, the focus was also on better compensation, the provision of adequate housing, and the acquisition of 300 additional cars, in order to facilitate home visits in rural areas.^4^ The position of GPs within policlinics was also to be improved, as many GPs working in policlinics felt subservient to doctors of other specialties. What did not succeed until the end of the GDR, however, was the establishment of chairs of general practice at one of the East German medical faculties. As a result, medical students still had little contact with general practice. This deficit, however, was not specific to the GDR. It was not until the second decade of the twenty-first century that general practice gained more visibility in the (now all-German) medical school curriculum.

## The Society for General Practice of the GDR

When the status of practical doctors was called into question in the GDR in the 1950s, some committed country doctors sought to improve networking not only among themselves but also with colleagues outside the GDR in order to strengthen their position. Thus, several doctors from the GDR (Brandt, Gärtner, Hohlfeld, Krause) together with colleagues from Austria, West Germany and Switzerland participated in the founding of the International College of General Practice in Vienna in 1959, which in 1964 became the Societas Internationalis Medicinae Generalis (SIMG) [2, 20]. The driving force behind this foundation was Robert N. Braun (1914–2007), a country doctor from Austria and tireless researcher in general practice. Because of his theoretical foundation of the field and his research on the frequency and classification of results of encounter in general practice [21, 22], Braun was highly respected in both parts of Germany. Also important was that Austria acted as a kind of neutral ground in the political system competition between East and West Germany, so that meetings between colleagues from both German states were much easier to set up there than in either East or West Germany. Moreover, in view of the GDR travel restrictions, East German physicians were more likely to be permitted to travel to congresses in Austria than in West Germany.

Pointing to the existence of the SIMG and their own prominent role in it, East German physicians worked persistently to establish a national society for general practice in the GDR. On the way there, they had to overcome resistance of colleagues from other disciplines as well as to take political hurdles. Since the SED was reluctant to allow any social or professional associations outside the party, the foundation of medical societies was a difficult task [23]. From the early 1960s on, establishing a new association was only possible under the umbrella of the party loyal German Society for Clinical Medicine (*Deutsche Gesellschaft für klinische Medizin*). In any case, medical societies were subordinate to the authority and approvement of the Ministry of Health. In 1969, after a long and arduous process, a group of GPs submitted an official application for the foundation of a society for general practice [24].^5^ It was crucial that they had the support of Ludwig Mecklinger (1919–1994), who at the time was State Secretary in the Ministry of Health. Mecklinger was well acquainted with Herbert Knabe, as both had worked at the University of Greifswald for a time.^6^ Mecklinger’s benevolence towards general practice became an even more important asset when he was appointed Minister of Health in 1971.

After a complicated verification a provisional board of the society for general practice could be constituted shortly before the end of the year 1969—as part of the Society for Internal Medicine. The board was appointed by Mecklinger on a handshake. Finally, after official registration at the Ministry of Health the inaugural meeting of the GDR society for general practice (*Gesellschaft für Allgemeinmedizin der DDR, GAM*) took place in Weimar in May 1971. The members present confirmed Heinz Barten (Altefähr/Rügen) as chairperson by election. In 1973, Gerda Junghanns (Bleicherode) was elected chairperson of the GAM and held the position for the following 10 years.^7^ In the 1970s a GAM regional district society was formed in each of the 15 regional districts of the GDR. An important task of these bodies was to conduct and supervise the specialist examinations in general practice.

Since the existing medical journals in the GDR offered little room for articles on general practice, the GAM established their own communication medium in 1974—the newsletter of the Society for General Practice (*Mitteilungen der Gesellschaft für Allgemeinmedizin der DDR*); however, the newsletter could not keep up with the established journals in terms of layout and scientific standards. In the late 1980s, the Ministry of Health developed the idea of converting the renowned *Zeitschrift für ärztliche Fortbildung* (Journal for Continued Medical Education, founded back in 1904) into a journal of general practice.^8^ In the end, however, this plan was not implemented.

In 1988, the number of GPs working in the GDR had risen to 9500.^9^ At the same time, membership of the GAM had reached the number of 5000, meaning that more than every second GP was a member of the GAM.^10^ Each year, the GAM held about 30 educational events for physicians with a total of 2000–3000 participants.^11^ Already in 1984, Grethe et al. published a comprehensive standard work on general practice which was widely read in the GDR [25].

General practice as a discipline had finally reached a remarkable professional level in the GDR, but there were two sides to the coin. It was bitter for many of the younger generation of East German GPs that before 1990 they hardly got the chance to set up their own practice. The official acceptance of general practice as a specialty had been accompanied by the further reduction of single doctor offices. In 1989, the last year of the GDR, the SED had almost reached its goal. While the total number of physicians had risen to just over 40,000, only 340 of them or less than 1% still had their own practice [9].^12^ The GDR Society of General Practice dissolved with the fall of the GDR and its health system. In 1991, it merged with the German College of General Practitioners and Family Physicians, the former West German society, which until then never had as many members as the East German society.

## Conclusion

The birth and growth of general practice as a specialty in the GDR resulted from the work of deeply committed physicians who, under difficult political circumstances, worked tirelessly in their field and pushed for its professionalization. The GDR healthcare policy favored a centrally organized state-driven health sector. This had a strong impact both on the medical profession and on medical practice. The traditional form of freelance work in an own practice was strongly curtailed. The socialist healthcare system saw policlinics as the best form of outpatient care. Besides the restraints for office-based doctors general practice in the GDR was consolidated and professionalized as a medical discipline. In the 1960s standards of training and qualification were defined to become an approved general practitioner. Compared to West Germany, for example, this was achieved quite early. The foundation of the GAM, the GDR Society of General Practice, in 1971 strengthened the position of the specialist field among other medical disciplines. It also facilitated networking among East German GPs and those from other countries; however, it did not lead to a better institutionalization at the medical faculties. The academic representation of general practice was limited to the Academy of Postgraduate Medical Education in East Berlin where two professorships of general practice existed since 1967.

The establishment of general practice as a specialty also seemed to have been facilitated by economic considerations, since it had become clear to SED health functionaries that primary care provided by GPs was cheaper and more cost-efficient than early specialist care. During the 1980s the GDR’s health system fell behind due to poor conditions of the buildings, outdated technical equipment and a growing shortage of medical supply and pharmaceuticals [26]. By the end of the 1980s, even a private telephone connection was not a matter of course for GPs. In Dessau, for example, only half of the GPs could be reached by telephone outside their practice; in Merseburg, only one in four could be reached at home.^13^ Nevertheless, the approximately 9,500 GPs, in whatever institution, continued to maintain and ensure preventive, prophylactic and long-term healthcare. What most of the GPs in the GDR had missed over the years was, among other things, the possibility to attend congresses in western countries or even to travel there privately [27]. There is a need for further research to compare the developments in the GDR described here not only with those in West Germany, but also with those in the socialist countries of Eastern Europe at that time.

## Footnotes

1 From 1972 on, general medicine as a specialist designation was only a voluntary qualification in West Germany, not necessary to set up one’s own doctor’s office. Since this specialist training was also little structured, a uniform qualification was not given. A compulsory specialist training of 5 years was introduced only in the 1990s after the German reunification to meet the East German standard.

2 Interview with Dr. Konstantin Kuminek, Bad Bibra, Germany, conducted by FB, September 2, 2019.

3 See “Vorlage zur Verwirklichung des Hausarztbeschlusses”, 19.4.1989, Bundesarchiv (BArch) Berlin, DQ 1/13253.

4 See “Maßnahmeplan zur weiteren Entwicklung der hausärztlichen Betreuung”, draft from April 15, 1988, as well as letter Mecklinger to Seidel, March 9, 1988, BArch Berlin, DQ 1/13263. In order not to upset the socialist economic plan the cars had to be taken from the quota intended for the general population.

5 Application to the Ministry of Health to form a Society for General Medicine in the GDR, headed by K. Scheidler, A. Hügel, supported by Prof. Dr. Bast, Dr. Brandt, Prof. Dr. Knabe, Prof. Dr. Igel, Dr. Junghanns, Dr. Kopseel, Dr. Kuminek, Dr. Glöckner Dr. Mitzscherling, Dr. Meumann. May 20, 1969. Vorlass Dr. Konstantin Kuminek, Institute for the History of Medicine, TU Dresden.

6 Interview with Dr. Konstantin Kuminek, Bad Bibra, Germany, conducted by FB, October 5, 2020.

7 Interview with Dr. Konstantin Kuminek, Bad Bibra, Germany, conducted by FB, September 4, 2019.

8 See “Maßnahmeplan zur weiteren Entwicklung der hausärztlichen Betreuung”, draft from April 15, 1988, BArch Berlin, DQ 1/13263.

9 Übersicht zum Ärztebestand in der DDR (31.12.1988), Fachrichtung Allgemeinmedizin, BArch Berlin, DQ 1/13253.

10 Interview with Dr. Konstantin Kuminek, Bad Bibra, Germany, conducted by FB, October 5, 2020.

11 G. Borgwardt: “Informationsmaterial der GAM zum Kongress 1989”, BArch Berlin, DQ 1/13254.

12 Among these few remaining self-employed doctors were not only GPs but also surgeons, internists and gynecologists. Personal communication Dr. Konstantin Kuminek, Bad Bibra, Germany, to FB, July 21, 2022.

13 Letter and statistical table from Heuschkel, district physician of Halle (Saale), to Harig, Deputy Minister of Health, December 13, 1988, BArch Berlin, DQ 1/13248.
